# Heart Rate Response to Blood Pressure Variations: Sympathetic Activation versus Baroreflex Response in Patients with End-Stage Renal Disease

**DOI:** 10.1371/journal.pone.0078338

**Published:** 2013-10-04

**Authors:** Dan Sapoznikov, Michal Dranitzki Elhalel, Dvora Rubinger

**Affiliations:** Nephrology and Hypertension Services, Department of Medicine, Hadassah Hebrew University Medical Center, Jerusalem, Israel; University of Otago, New Zealand

## Abstract

**Background:**

Continuous systolic blood pressure (SBP) and interbeat intervals (IBI) recordings reveal sequences of consecutive beats in which SBP and heart rate change in opposite direction, representing negative feedback baroreflex mechanisms, as well as sequences in which SBP and heart rate change in the same direction (non-baroreflex), believed to represent feedforward control mechanisms. The present study was undertaken to assess the relationship between baroreflex and non-baroreflex sequences in end stage renal insufficiency.

**Methodology/Principal Findings:**

Continuous beat-to-beat SBP and IBI monitoring was performed in patients on chronic hemodialysis (HD, n=72), in age-matched patients after renal transplantation (TX, n=41) and healthy (control) individuals (C, n=34). The proportion of baroreflex and nonbaroreflex episodes and the ***b*** coefficients (the regression line slope of SBP-IBI correlation) were determined using a newly developed 1 minute sliding window method, the classical sequence technique and the "Z" coefficient method. Analysis using the 1 minute sliding window showed an increased proportion of baroreflex episodes in controls and HD, and predominance of nonbaroreflex episodes in TX. An increased proportion of nonbaroreflex episodes in TX patients relative to HD was also revealed by the "Z" method. Baroreflex and nonbaroreflex ***b*** coefficients obtained by all methods were markedly decreased in HD. This alteration was reversed at least partly in TX. In HD, both baroreflex and nonbaroreflex ***b*** coefficients were inversely correlated to age and CRP levels; in TX, the nonbaroreflex ***b*** coefficient was influenced by the type of calcineurin inhibitor.

**Conclusion/Significance:**

Renal status affects the contribution of baroreflex and nonbaroreflex mechanisms and the strength of SBP-IBI relationship. The predominant contribution of nonbaroreflex mechanisms in TX may be suggestive of enhanced central sympathetic control. Our data may be relevant for understanding of the pathogenesis and selection of appropriate treatment of post-transplant hypertension.

## Introduction

Blood pressure and heart rate changes are related through various nervous and hormonal mechanisms. Sympathetic nervous system has a major role in arterial blood pressure control. Sympathetic outflow increases arterial pressure via vasoconstriction (feedforward) while elevations in blood pressure suppress sympathetic outflow via baroreflex (feedback) mechanism. Baroreflex activity is characterized by an inverse relationship between systolic blood pressure (SBP) and heart rate. Continuous recordings of systolic blood pressure (SBP) and interbeat intervals (IBI) reveal time-sequences of spontaneously occurring consecutive beats in which blood pressure and heart rate change in opposite direction (i.e. increased SBP with increased IBI, or decreased SBP with decreased IBI). These sequences are considered to be an expression of the negative feedback mechanisms of baroreflex origin [1,2]. In many episodes, however, heart rate is directly related to SBP, for instance increased blood pressure with tachycardia (decreased IBI), or decreased blood pressure with bradycardia (increased IBI). Theses episodes are defined by some investigators as "non-baroreflex" sequences [3,4]. The physiological significance of the latter is not clear. While non-baroreflex episodes are considered by many investigators to reflect feedforward mechanisms of centrally activated sympathetic control of arterial pressure [1,2,4,5], an alternative interpretation claims that they represent perturbative events of blood pressure changing following IBI modifications according to Starling law and arterial distensibility [6]. The sympathetic contribution to the generation of nonbaroreflex sequences was supported by animal experiments and human studies. Feedforward regulated mechanisms were proposed to play a role during short and long term cardiovascular regulation, in conditions such as different sleep stages, essential hypertension, and myocardial vascularization after coronary ischemia [7-12]. Recently, feedforward regulated mechanisms were also suggested to play a role in the generation of hypertensive episodes during hemodialysis procedures [13].

Sequence analysis is a popular method of identifying both baroreflex and non-baroreflex sequences [14]. The sequence method is based on simultaneously increased or decreased SBP (1mmHg change) and IBI (6 msec change) for short sequences (at least 3 beats). Such sequences are rarely of long duration. These transient SBP and IBI changes, however, may be different from long term characteristics of SBP-IBI relationship.

Spectral analysis of SBP and IBI fluctuations with calculations of α coefficient is a frequently used method to estimate baroreceptor sensitivity (BRS). This technique, however, cannot discriminate between negative and positive feedback components, i.e. is not able to identify nonbaroreflex mediated activity [14,15].

The estimation of "Z"-index is based on the computation of the statistical dependence between SBP and heart rate values with the Z coefficient [16]. This method allows for identification, during spontaneous cardiovascular activity, of couples of SBP and heart rate values linked to the baroreflex stimulation or to direct central (feedforward, nonbaroreflex) control [16]. While several studies used the Z-method to assess baroreflex sensitivity in humans [15,17], the contribution of nonbaroreflex mediated activity was evaluated only in animal studies [16].

The aim of the present paper was to analyze the interrelation between baroreflex and nonbaroreflex sequences in the larger context of end stage renal insufficiency. Within this purpose, we developed a sliding window methodology to estimate SBP-IBI correlation for longer periods of time. SBP-IBI correlations calculated during one-beat overlapping 1 minute epochs were compared with estimations obtained using the traditional sequence method (time domain analysis), the frequency domain analysis and the Z method. We studied patients with end- stage renal disease on chronic hemodialysis and after renal transplantation as compared with normal individuals.

## Patients and Methods

Continuous beat to beat blood pressure recordings were obtained in 72 non-diabetic patients on chronic hemodialysis (HD), in 41 age-matched patients after renal transplantation (TX) and in 34 aged-matched normal individuals (controls, C). The healthy individuals were recruited among hospital staff and medical students. The study was approved by Hadassah-Hebrew University Medical Center Ethics Committee for Clinical Research. The patients signed an informed consent form before taking part in the study.

Patients with chronic atrial fibrillation or frequent ventricular premature beats, debilitating illness and frequent hospital admissions, permanent pacemakers and severely decreased radial pulses following multiple vascular accesses surgical interventions were excluded from the study. Antihypertensive drugs were stopped 12-14 hr before the study. Patients’ clinical and demographic characteristics are presented in Table 1.

**Table 1:** Demographic and clinical data of controls (C), of hemodialysis (HD) and transplanted (TX) patients.

	C	HD	TX
n	34	72	41
Age (years)^	53 + 11	55 + 16	53 + 11
Gender (M/F)	15/19	46/26*	28/13**
History of hypertension	-	62 (86.1%)	35 (85.4%)
Ischemic heart disease	-	34 (47.2%)	11 (26.8%)^a^
History of smoking	-	30 (41.7%)	10 (24.4%)^b^
Hyperlipidemia	-	45 (62.5%)	26 (63.4%)
Antihypertensive medication	-	48 (66.7%)	28 (68.3%)
Nitrates	-	10 (13.9%)	3 (7.3%)
Calcium blocking agents	-	26 (36.1%)	12 (29.3%)
Vasodilators	-	11 (15.3%)	5 (12.2%)
ACE inhibitors	-	14 (19.4%)	12 (29.3%)
Angiotensin receptor blockers	-	4 (5.6%)	8 (19.5%)^c^
Beta blockers	-	36 (50.0%)	18 (43.9%)
Calcineurin inhibitors:			
Cyclosporin	-	-	12 (29.3%)
Tacrolimus	-	-	28 (68.3%)
mTOR inhibitors	-	-	2 (4.9%)
SBP (mmHg)^	126 ± 15	136 ± 24***	126 ±18^a^
DBP (mmHg)^	72 ± 10	75 ± 13	70 **±** 10^d^
PP (mmHg)^	54 ± 10	61 ±18****	56 ± 13
IBI (msec)^	822 ±110	820 ±130	811 ±123

p * 0.044; ** 0.030; *** 0.015 **** 0.009 vs. controls. ^a^ 0.026; ^b^ 0.049; ^c^ 0.025; ^d^ 0.047 vs. HD; **^** mean ± SD. ACE- Angiotensin converting enzyme; mTOR- mammalian target of rapamycin; SBP-systolic blood pressure; DBP- diastolic blood pressure; PP-pulse pressure.

The hemodialysis patients (at least half year on maintenance therapy, duration [median (interquartile range)] 0.84 (2.10) yr) were dialyzed three-four times weekly, 3.5 to 5 hours each time. Hemodialysis was performed using a polyamide high flux dialyzer (1.3 m^2^) and the mean urea reduction rate (% URR) in all patients was 65-75%. The duration of the recordings was 3.5 to 5 hr for the dialysis patients and 45 min to 1.5 hr for controls and transplanted patients. In hemodialysis patients, the studies were performed on a midweek dialysis day. The mean values of indices obtained over the extended time (several hours), were considered to be most representative for dialysis patients in whom significant fluctuations in fluid balance and metabolic status occur at least 3 times weekly. All dialysis patients were treated with recombinant erythropoietin and phosphate binders (calcium carbonate, lanthanum carbonate and sevelamer hydrochloride) as needed.

The studies in transplanted patients (duration of transplantation (median [interquartile range] 0.786 [6.77]) were performed at least 3 months after recovery of renal function. Thirty six patients have been in chronic hemodialysis before transplantation; pre-emptive transplantation was performed on the remaining 6 patients. The plasma creatinine level (mean ± SD) was 115±31 µmol/l. Their maintenance immunosuppressive therapy included corticosteroids, mycophenolate mofetil or azathioprine, calcineurin inhibitors or mTOR inhibitors (Table 1). Mean (trough) blood levels of cyclosporine and tacrolimus were 65-105 ng/ml and 6-8 ng/ml respectively.

Biochemical data and CRP (C- reactive protein) levels were obtained in all dialysis and transplanted patients.

Blood pressure and inter-beat intervals were measured noninvasively by Finometer 1.10 (FMS, Finapres Medical Systems BV, Arnhem, The Netherlands). Continuous beat to beat recording of the finger arterial pressure waveform was performed using the volume-clamp method of Penaz [18] and the Physiocal criteria of Wesseling, as previously described [19]. The finger cuff was wrapped on the middle phalanx of patient’s middle finger. A height correction unit was attached to adjust for finger height changes during the recording. An upper arm cuff unit was also attached and used to calibrate the systolic pressure for calculating the reconstructed brachial pressure from the finger pressure.

The raw data were downloaded into a PC and analyzed by the BeatScope Software (TNO TPD Biomedical Instrumentation Amsterdam, The Netherland) and our specifically designed programs. The BeatScope software performs beat to beat analysis of the raw finger arterial pressure and uses filtering and level correction to calculate reconstructed brachial pressures from finger pressures. The resulting data files containing beat to beat values of hemodynamic variables such as inter-beat intervals (IBI), systolic (SBP), diastolic and mean blood pressures, were used for further processing.

### SBP-IBI relationship during overlapping 1 minute time windows

**Figure 1:**
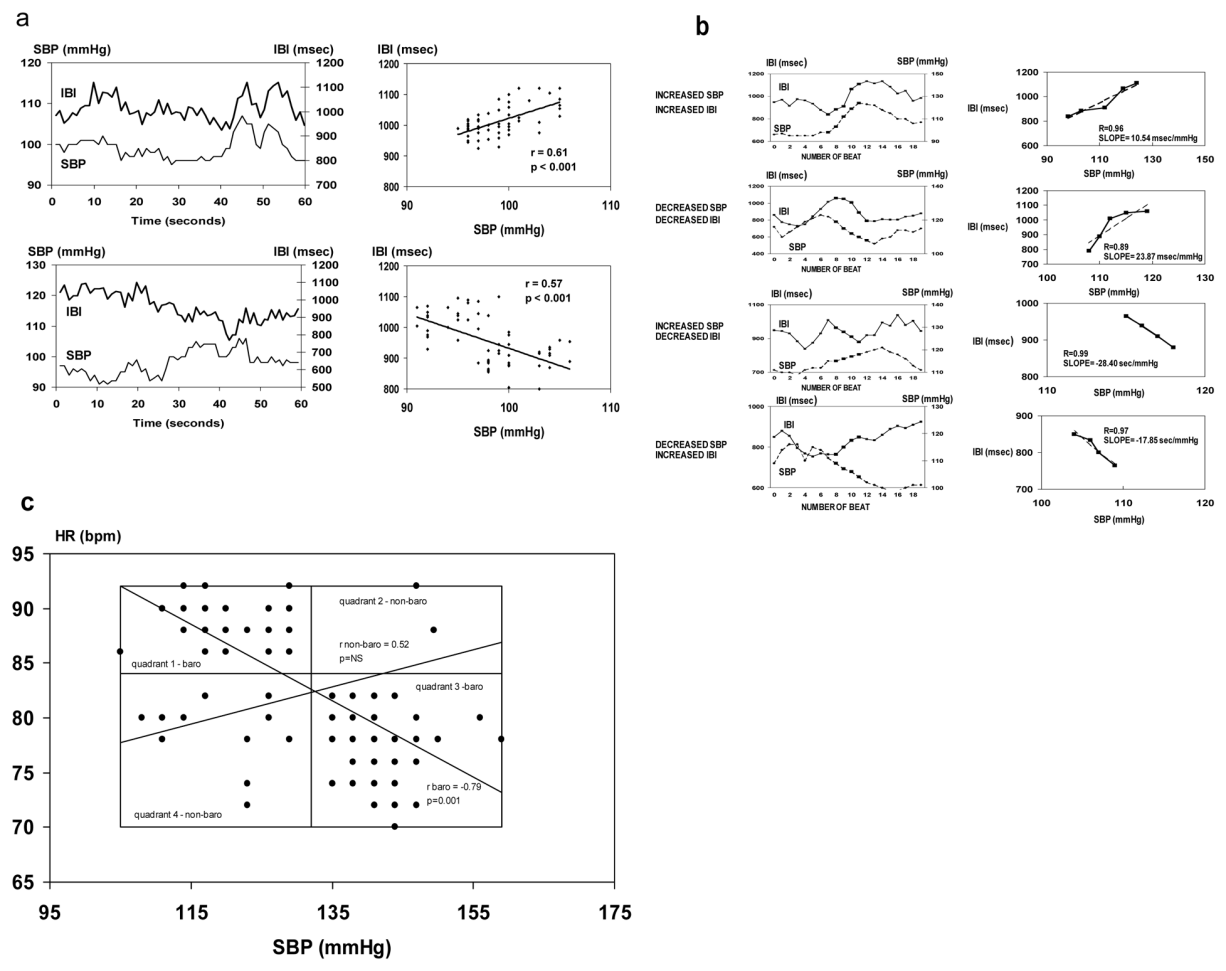
Baroreflex and non-baroreflex function determined by three methods. SBP-systolic blood pressure; IBI-interbeat interval; HR- heart rate. (**a**) The sliding window method: SBP and IBI tracings and their correlations during a baroreflex episode (SBP and IBI change in the same direction, upper panel) and during a non-baroreflex episode (SBP and IBI change in different directions, lower panel). (**b**) The sequence technique: SBP and IBI tracings and their correlations during baroreflex episodes (SBP and IBI change in the same direction, increased or decreased SBP, upper panels) and during nonbaroreflex episodes (SBP and IBI change in opposite directions, increased or decreased SBP, lower panels). (**c**) The "Z" method: Three dimensional histogram for couples of SBP and HR. The modal class (the maximal, most frequent HR-SBP histogram value) was taken as a set point of spontaneous activity. The SBP-HR pair classes in quadrant 1 (low SBP associated with high HR values) and in quadrant 3 (high SBP associated with low HR values) are representative of baroreflex episodes, while quadrants 2 (low SBP with low HR) and 4 (high SBP with high HR) represent non-baroreflex episodes. Regression lines and correlation coefficients are shown for baroreflex episodes (quadrants 1 and 3) and for nonbaroreflex episodes (quadrants 2 and 4).

Overlapping one minute time windows at one beat steps were selected for analysis of beat to beat IBI-SBP relationship during the whole recording session for each patient. The correlation coefficient and the slope of the regression line between IBI and SBP were calculated for each one minute epoch. One minute epochs with correlation coefficients higher than 0.5 were selected for analysis (Figure 1a). The percentages of epochs with positive or negative correlation and their respective average regression coefficients were then calculated for every patient. Median and interquartile values of the percentages (% episodes) and regression coefficients (slopes, ***b*_*sl*_**) were calculated and compared for Control, HD and TX groups. Positive ***b*_*sl*_** were considered to be representative of the sensitivity of the baroreflex-mediated changes, while negative ***b*_*sl*_** were considered to be representative of the sensitivity of those mediated by nonbaroreflex mechanisms.

Although the latter method does not distinguish between increased or decreased SBP, a good estimate is obtained for distinguishing between direct and inverse correlation of SBP-IBI. Periods longer than 1 minute did not show good correlations due to the non-stationary character of SBP and IBI changes. Overlapping periods with one beat steps are beneficial to this analysis since every stretch of data is covered and epochs with good correlations are not missed.

### The sequence method

Beat to beat systolic blood pressure (SBP) and inter-beat intervals (IBI) were scanned for sequences of SBP increase or decrease (ramps) and for sequences in which SBP and IBI either increase or decrease for at least 3 beats. The thresholds for IBI and SBP beat to beat change were 6 msec and 1 mmHg respectively.

Sequences were evaluated within four categories of slope: 1. increased SBP-increased IBI, 2. decreased SBP-decreased IBI, 3.increased SBP-decreased IBI, and 4. decreased SBP-increased IBI. Representative examples of the four categories of sequences are demonstrated in Figure 1b.

The derived parameters were: 1. the percent of sequences in each category out of the total number of sequences (%b1, %b2, %b3 and %b4), and, 2. the slope of the regression line between IBI and SBP for at least 3 beats for each category (b_1_, b_2_, b_3_ and b_4_). Patients’ mean slope was calculated for all valid sequences in which the correlation coefficient (R) was greater than 0.85. b_1_ and b_2_ were the regression line slopes of sequences where both SBP and IBI increase or decrease were considered as due to baroreflex response. b_3_ and b_4_ were the regression line slopes of sequences in which SBP and IBI changed in opposite directions and were considered as due to non-baroreflex episodes. Accordingly, b_1_ and b_2_ were combined as the baroreflex slope ***b*_*seq*_**, while b_3_ and b_4_ were combined as the non-baroreflex slope ***b***_seq_.

In patients with extremely low heart rate or blood pressure variability, sequences of SBP and IBI increase or decrease meeting the above criteria were not detected. Therefore, not all patients were included in slope calculations by either of the methods.

### Frequency domain analysis

Power spectrum analysis was performed using the Welch periodogram method after detrending to remove slow non-periodic variations, as previously described [20,21]. Resampling of SBP and IBI signals was performed with a sampling rate of 2 Hz. Power spectrum amplitudes of SBP, IBI, and their cross spectrum were obtained for noise-free five minute chosen epochs. Every five-minute interval contained 600 data points. The Welsh periodogram was calculated from 11 overlapping segments of 100 samples each with 50 samples overlapping. A Hamming window was applied on each segment before power spectrum analysis. Frequency domain variables were calculated in two frequency bands: the low frequency range (LF 0.04-0.15 Hz) and the high frequency range (HF 0.15-0.40 Hz).

The alpha coefficient (α) was defined as the square root of the ratio between the average power spectral densities of IBI and SBP in the frequency band:

$$alpha(\alpha )=\sqrt{\frac{\bar{Py(f)}}{\bar{Px(f)}}}$$

where $\bar{Px(f)}$ and $\bar{Py(f)}$ are the average power spectral densities over the frequency range of SBP and IBI, respectively.

The values of the above parameters were averaged for all epochs chosen for an examined subject. The α coefficient was averaged for epochs with a coherence above 0.5.

### The "Z" method

Estimation of the "Z" index is a statistical method to assess the spontaneous cardiac baroreflex developed by Ducher et al [16,17]. A three dimensional histogram for couples of SBP and heart rate (HR) was calculated. Data were pooled in intervals of 3 mmHg for SBP and 2 beats/min for HR. The modal class was defined as the maximal, most frequent IBI-SBP histogram value and was taken as a set point of spontaneous activity. Four quadrants were defined centered on the modal class. The SBP-HR pair classes in quadrant 1 (low SBP associated with high HR values) and in quadrant 3 (high SBP associated with low HR values) were reported to be related to baroreflex episodes. The same analysis was performed for quadrants 2 (low SBP with low HR) and 4 (high SBP with high HR), representing non-baroreflex episodes. For each SBP-IBI couple a coefficient of statistical dependence (Z) was calculated as follows:

$$\text{Z }\left(\text{SBP, HR}\right)\text{ = }\left[\text{N }\left({}_{\text{SBP, HR}}\right){\text{/N}}_{\text{SBP}}{\text{– N}}_{\text{HR}}{\text{/N}}_{\text{T}}\right]\text{/ }\left[{\text{1 – N}}_{\text{HR}}{\text{/N}}_{\text{T}}\right]$$

where N_T_ represents the total number of beats recorded, N _(SBP, HR)_ the number of occurrences of (SBP,HR) couples and N_SBP_ and N_HR_ the number of occurrences of each class of SBP and IBI respectively. The number of beats in each quadrant was counted for couples with more than 10 beats in each interval and the percent from the total count of beats in the four quadrants was calculated (Figure 1c).

The linear regression between selected SBP and HR values were calculated for each couple with more than 10 occurrences and Z > 0. In order to emphasize couples with frequent occurrence and a strong dependence, each couple was weighted by P(SBP,HR) x Z(SBP,HR), where P(SBP,HR) represents the estimated conditional probability of observing HR and SBP, while Z(SBP, HR) was defined above [17]. The absolute value of the slope (*b*_*z*_) of the regression line in quadrants 1 and 3 was considered as an index of the spontaneous baroreflex sensitivity (BRS), while the value of the slope of the regression line in quadrants 2 and 4 was considered to be an index of strength of non-baroreflex relationship if the linear adjustment was significant (p<0.001).

### Statistics

Demographic and clinical categorical variables were compared by χ^2^ test. Age, SBP, DBP and PP (mean and standard deviation) were compared using Student’s *t* test. ***b*** indices, LF α and HF α are given as medians and interquartile ranges. Mann-Whitney U test was performed to compare ***b*_*sl*_**, ***b*_*seq*_** and ***b*_*z*_** between groups and between tertiles of hemodialysis vintage. Correlations of the absolute value of the ***b*_*sl*_** with ***b*_*seq*_** and ***b*_*z*_**, with age and with LF α and HF α coefficients were performed by Pearson correlation and linear regression analysis.

### Definitions of clinical variables

Ischemic heart disease was defined by documentation of prior myocardial infarction or of coronary interventions, the presence of significantly abnormal Q waves on a 12 lead electrocardiogram, symptomatic angina pectoris or a thallium perfusion scan suggestive of myocardial ischemia. Patients with high triglyceride or/and high LDL cholesterol levels were considered to be hyperlipidemic.

## Results

### Clinical data

Patients’ clinical data are depicted in Table 1. The number of women was lower in both HD and TX as compared with the control group. While a similar proportion of patients with a background of hypertension or hyperlipidemia was noted in HD and TX, more smokers and more patients with a history of ischemic heart disease were found in HD group. HD patients were also more hypertensive than controls and TX; in the latter, blood pressure was within the same range as in controls. The native original kidneys were present in all HD and TX patients.

### Distribution of baroreflex and and nonbaroreflex episodes assessed by the 1 minute epoch method, by the sequence technique and by the "Z" coefficient method

Representative examples of baroreflex and nonbaroreflex sequences obtained by the 1 minute epochs tracings, the sequence method and the "Z' coefficient method, with direct and inverse correlations of IBI-SBP, are shown in Figure 1a, 1b and 1c, respectively.

**Figure 2:**
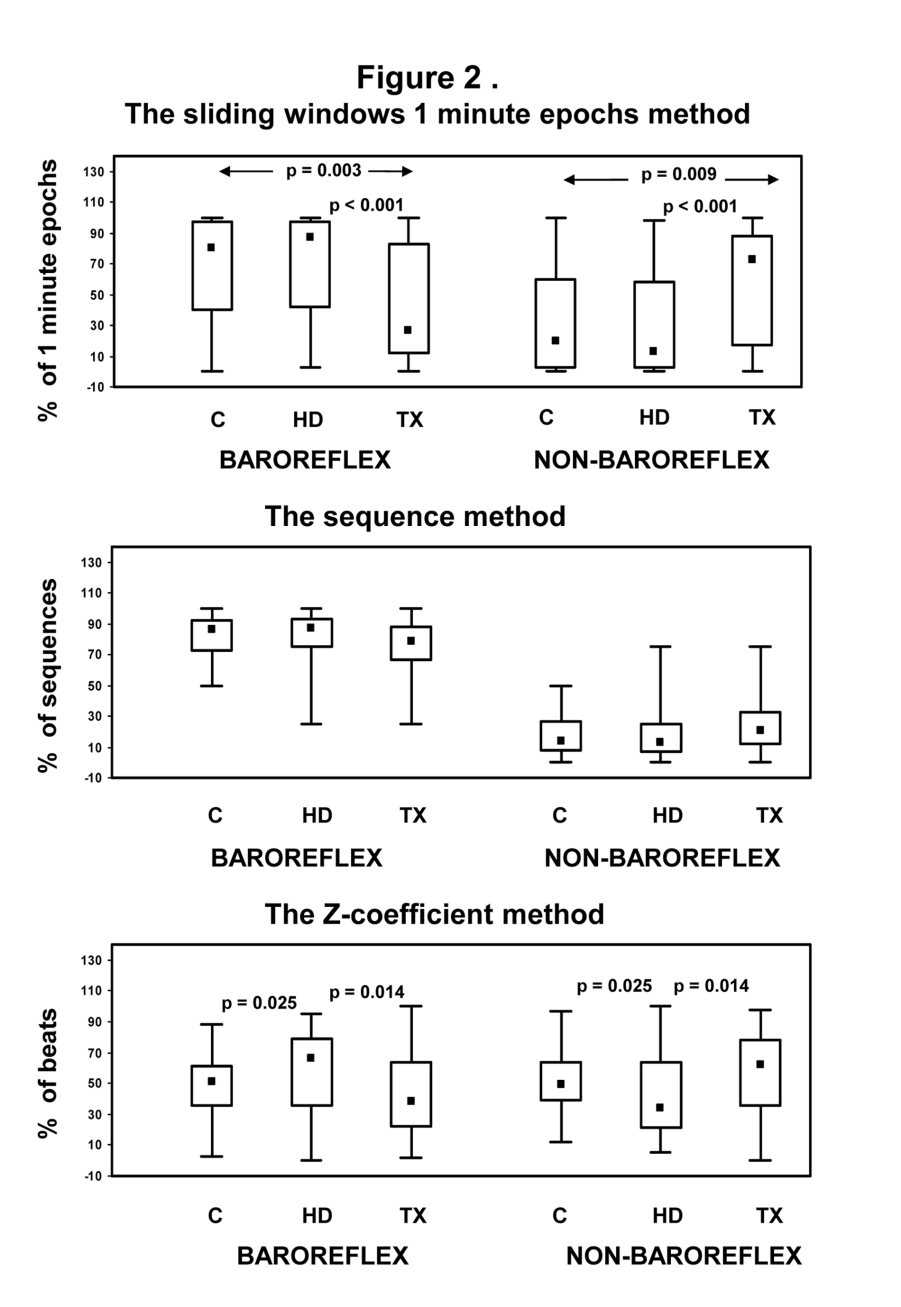
Distribution (%) of baroreflex and non baroreflex episodes in control (C), in hemodialysis (HD) and in transplanted (TX) patients determined by three methods.

The proportion (% of total) of baroreflex and non- baroreflex episodes obtained by 1 minute epochs (sliding-window) method, by the sequence technique and by the "Z" method are depicted in Figure 2. The proportion (% of 1 minute epochs) of baroreflex episodes detected by the sliding windows 1 minute epochs analysis was significantly higher in Controls and HD than in TX patients. In Controls and HD, the proportion of nonbaroreflex episodes was much lower than that of baroreflex episodes. In contrast, in TX patients the sliding window 1 min analysis revealed a marked increase in the proportion of nonbaroreflex episodes and a decrease in the proportion of baroreflex episodes (Figure 2, upper panel).

When the traditional sequence method was used, baroreflex sequences in which SBP and IBI were directly proportional, were more frequent, while the number of nonbaroreflex sequences in which SBP and IBI were inversely correlated was significantly smaller in all groups (Figure 2, middle panel). The proportion of baroreflex and nonbaroreflex episodes (% of total sequences) obtained by the sequence method was similar in control, in HD and in TX patients.

The proportion (% of beats) of baroreflex and non-baroreflex episodes detected by the Z method is shown in Figure 2, lower panel. In controls, the distribution of both types of episodes was similar. In HD patients, there was a significant increase in the proportion of baroreflex and a decrease in that of non-baroreflex episodes. In transplanted patients, the distribution of baroreflex and non-baroreflex episodes was similar to that of healthy controls.

**Figure 3:**
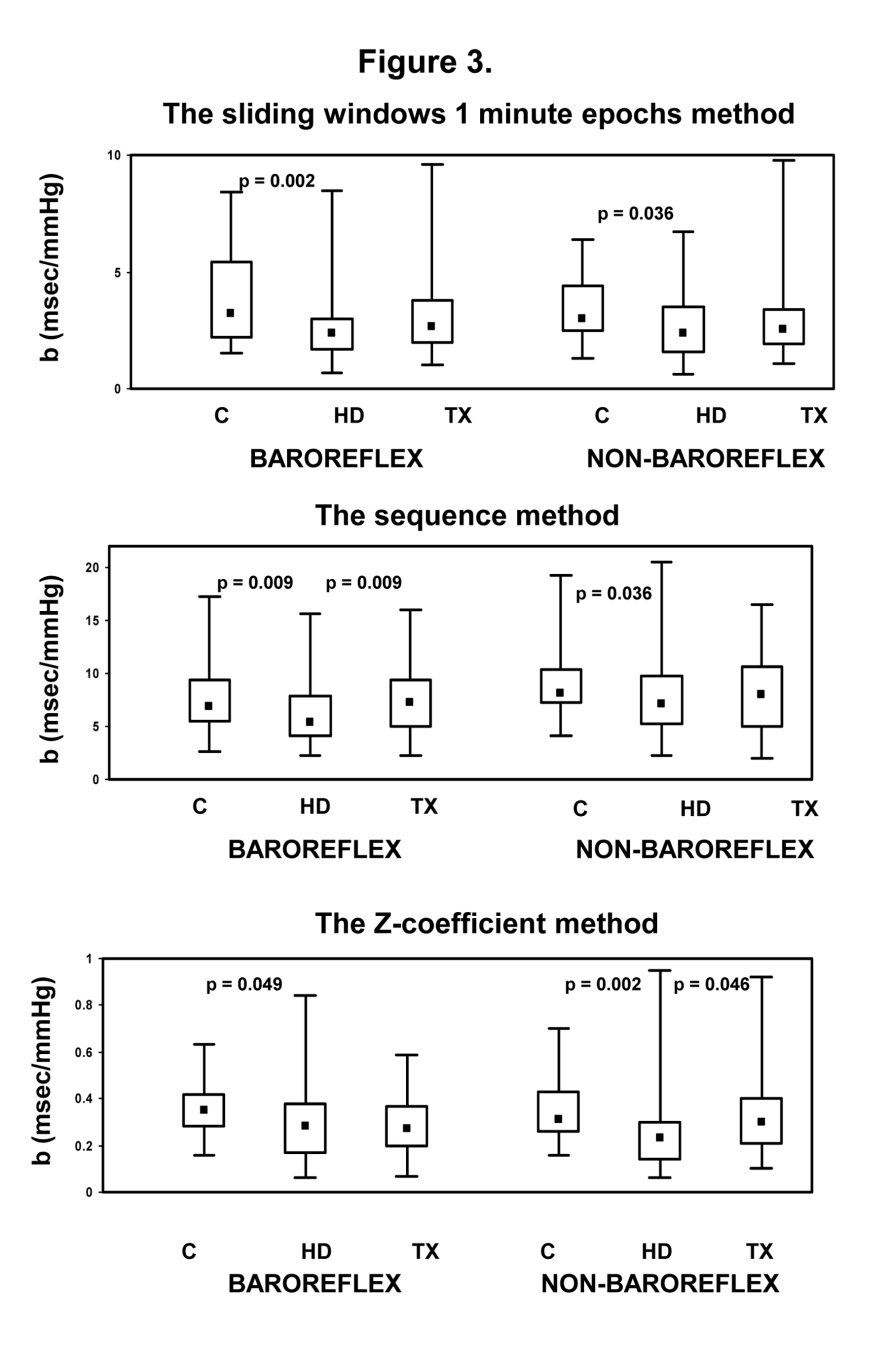
IBI-SBP regression slopes of baroreflex and nonbaroreflex episodes obtained by the 1 min sliding window analysis (*b*_*al*_), by the sequence method (*b*_*seq*_), and by the "Z" technique (*b*_z_) in control (C, •), in hemodialysis (HD, ♦) and in transplanted (TX,■) patients.

The regression slopes of baroreflex- and nonbaroreflex episodes obtained by the three above methods (*b*_*sl*_ obtained by the 1min sliding window analysis, *b*_*seq*_, obtained by the sequence method and *b*_z_, obtained by the Z technique) from the three patient groups are shown in Figure 3. All slopes are given as absolute values.

Significant decrease in the baroreflex slopes (*b*_*seq*_, *b*_sl_ and *b*_z_) in HD patients as compared to Controls were detected by all methods. The slopes of non baroreflex episodes were also significantly decreased in HD patients. A trend to an increase in the slopes of both baroreflex and nonbaroreflex episodes after TX was noted using all methods; in these patients, the increases in the baroreflex slope (*b*_*seq*_) using the sequence method (Figure 3, mid-panel) and an increase in the nonbaroreflex slope(*b*_z_) using the "Z" method (Figure 3, lower panel) were statistically significant.

**Figure 4:**
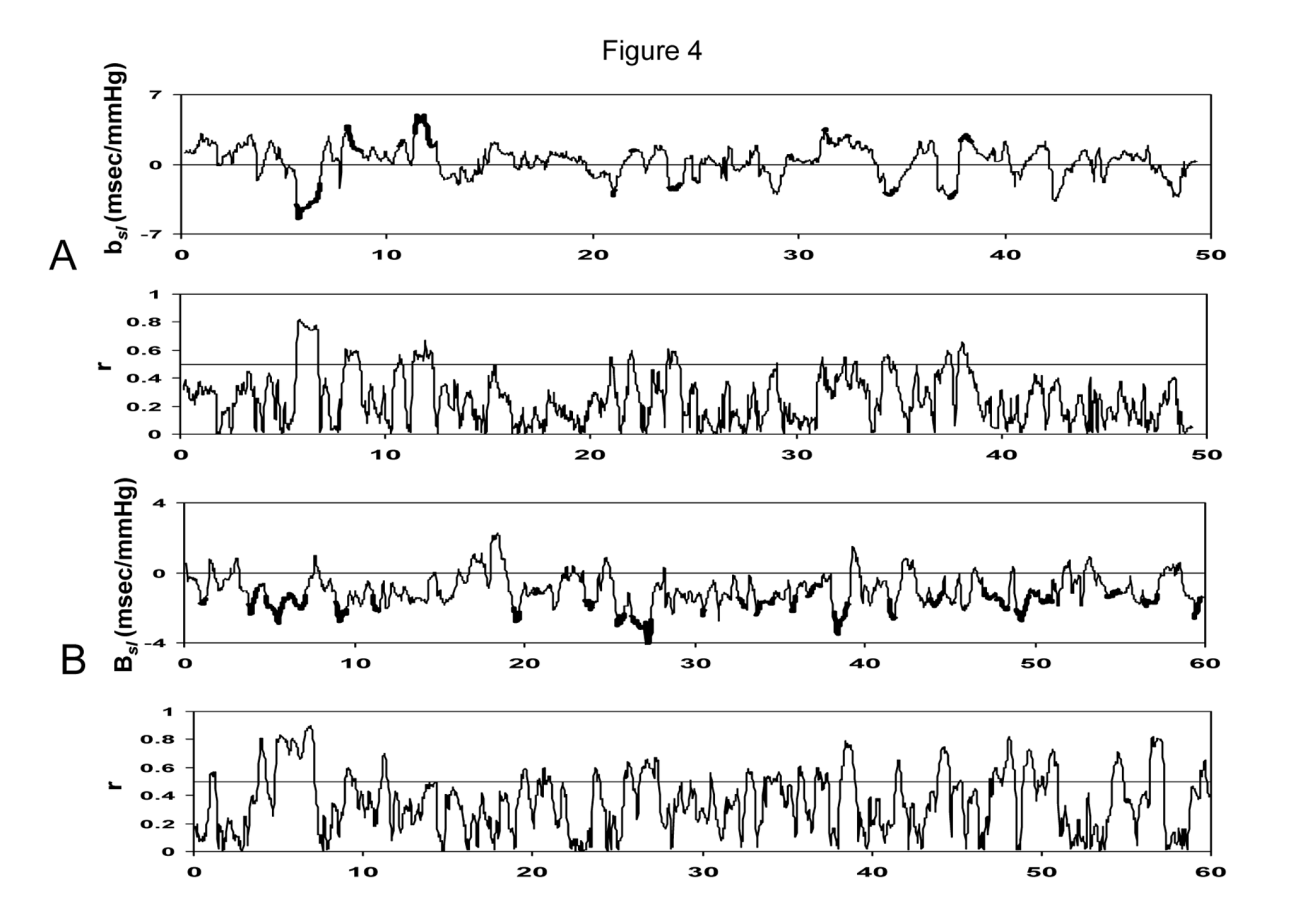
Representative tracings of *b*_*sl*_ (the slope of 1 min IBI-SBP regression line assessed by 1 min sliding window method), and r-the correlation coefficient as a function of time during the whole session of the recording in a control (upper panels, A) individual and in a transplanted patient (lower panels, B). The bold portions of ***b*_*sl*_** tracings represent epochs with a correlation coefficient (r) between IBI and SBP greater than 0.5. In the control patient’s tracing, 55% of episodes in such epochs were baroreflex with a positive *b*_*sl*_, while the remaining 45% were non-baroreflex with a negative *b*_*sl*_. A similar tracing for a transplanted patient is shown in Figure 4, lower panel. In this patient, all sequences (100%) were nonbaroreflex episodes.

Representative tracings of ***b*_*sl*_** – the slope of the IBI-SBP regression line assessed by 1 min sliding window method, and r-the correlation coefficient as a function of time during the whole session of the recording in a control (upper panel, A) individual and in a transplanted patient (lower panel, B) are shown in Figure 4. In the control patient’s tracing, 55% of detected episodes in the analyzed epochs (those with the correlation coefficient (r) between IBI and SBP >0.5) were baroreflex episodes with a positive *b*_*sl*_ coefficient, while the remaining 45% with a negative *b*_*sl*_ coefficient were non-baroreflex. A similar tracing for a transplanted patient is shown in Figure 4, lower panel. In this patient, all sequences (100%) were nonbaroreflex episodes.

### Correlations of baroreflex and nonbaroreflex slopes determined by 1 min sliding window (b_sl_) with those obtained by other methods

**Figure 5:**
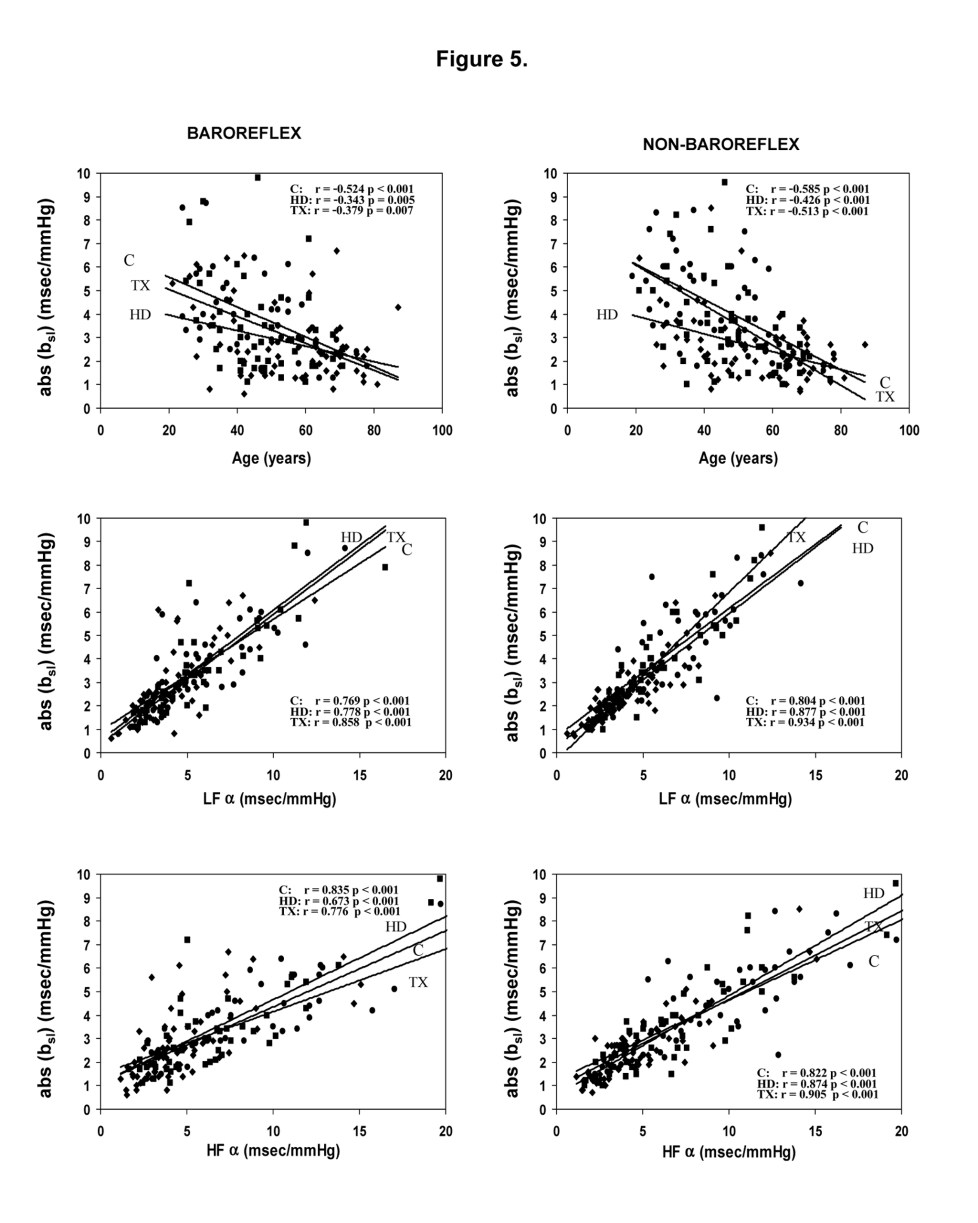
Correlations of baroreflex and nonbaroreflex *b*_*sl*_ slopes (absolute values) with age and with LFα and HFα indices in control (C), in hemodialysis (HD) and in transplanted (TX) patients.

Table 2 lists the correlations of the baroreflex and nonbaroreflex regression slopes determined by 1 min sliding window method (***b*_*sl*_**) with those obtained using the sequence (***b*_*seq*_**) and the "Z" (***b*_*z*_**) methods. ***b*_*sl*_** was strongly correlated to ***b*_*seq*_** both baroreflex and nonbaroreflex episodes in all patient groups and to a lesser extent to *b*_*z*_, especially for TX patients. In each of the three methods, the baroreflex slopes were significantly correlated to nonbaroreflex slopes for all patients groups (p=0.001 for ***b*_*sl*_*,b*_*seq*_**, and ***b*_*z*_**, in controls, HD and TX, respectively). Significant correlations of baroreflex and nonbaroreflex ***b*_*sl*_** slopes were also found with LFα and HFα coefficients, representative of both baroreflex and nonbaroreflex function determined by power spectrum technique (Figure 5).

**Table 2:** Correlation coefficients of baroreflex and nonbaroreflex slopes determined by 1 min sliding windows method (*b_sl_*) with those obtained by the sequence technique (*b_seq_*) and the "Z" method (*b_z_*), in control individuals (C), in hemodialysis (HD) and in transplanted (TX) patients.

		***b*_*sl*_** -*b*_*z*_	***b*_*sl*_**- ***b*_*seq*_**	***b_z_ -b_seq_***
**Baroreflex slopes**	C	0.804	0.819	0.702
		p=0.000	p=0.000	p=0.000
	HD	0.522	0.809	0.406
		p=0.000	p=0.000	p=0.001
	TX	-0.124	0.531	0.079
		p=0.573	p=0.001	p=0.713
**Non-baroreflex slopes**	C	0.568	0.556	0.407
		p=0.003	p=0.002	p=0.039
	HD	0.179	0.422	0.448
		p=0.218	p=0.001	p=0.002
	TX	0.605	0.624	0.266
		p=0.000	p=0.000	p=0.172

### Correlations of baroreflex and nonbaroreflex activity assessed by 1 min sliding window with clinical and laboratory data

In healthy individuals, but not in HD or TX patients, the percentage (%) baroreflex episodes was higher in men (95%) than in women (65%, p=0.007). Neither baroreflex nor non-baroreflex episodes were affected by age, basic renal or background diseases, biochemical data, antihypertensive or immunosuppressive drugs (not shown in figures). The effect of dialysis vintage on the proportion of baroreflex episodes in HD and TX patients is shown in Table 3. The patients were divided into tertiles according to the time spent in hemodialysis. In HD patients, the baroreflex contribution (% episodes) was inversely correlated to dialysis vintage. Over time, baroreflex episodes remained predominant in HD patients, but their relative contribution decreased with time (Table 3), while the relative contribution of nonbaroreflex episodes (not-shown in Table) increased. The effect of the dialysis vintage was assessed in only 35 of 41 transplanted patients, since 6 patients underwent preemptive transplantation. In these patients, the proportion of baroreceptor episodes was lowest in those who spent the longest time in dialysis (3^rd^ tertile, Table 3).

**Table 3:** Effect of hemodialysis vintage on the baroreflex contribution (% baroreflex episodes) in hemodialysis (HD) and in transplanted (TX) patients.

	Hemodialysis vintage in HD patients (range 0.11-20.98 y)		Hemodialysis vintage in TX patients (range 0.08-10.99 y)	
Patients	n=72		n=35	
Tertile	% Baroreflex episodes	p (vs.1^st^ Tertile)	% Baroreflex episodes	p (vs. 1st Tertile)
1	94 (16)		71 (70)	
2	68 (57)	0.024	27 (75)	0.311
3	86 (73)	0.034	13 (32)	0.049

The patients were divided into tertiles according to the time spent in hemodialysis.

***b*_*sl*_** slopes of both baroreflex and nonbaroreflex episodes in all study groups were markedly negatively correlated to age (Figure 5). In HD patients, significant negative correlations of ***b*_*sl*_** were also found with CRP level (r=-0.328, p= 0.005 for baroreflex and r=0.348, p= 0.004 for nonbaroreflex episodes, respectively). Similar correlations with age and CRP were also noted for ***b*_*seq*_** (data not given). There were no correlations of the ***b*** coefficients with other biochemical parameters.

### Effect of calcineurin inhibitors

Forty TX patients were treated with calcineurin inhibitors, the majority of them with tacrolimus (Table 1). There were no differences in the proportion of baroreflex or nonbaroreflex episodes, assessed by each of the three methods, with regard to the type of calcineurin inhibitor agent. The ***b*** coefficients (***b*_sl,_*b*_*seq*_** and ***b*_*z*_**) of baroreflex episodes were similar in patients treated with cyclosporine or tacrolimus. In nonbaroreflex episodes, ***b*_*sl*_**, ***b*_*seq*_** and ***b*_*z*_** (median (interquartile range)) were 3.10 (1.90), 8.85 (4.10) and 0.26 (0.17) in patients treated with cyclosporine and 2.20 (1.30), 7.10 (5.20) and 0.31 (0.20) in those treated with tacrolimus (p=0.013, 0.031 and NS, respectively.

***b*_*sl*_**, ***b*_*seq*_** and ***b*_*z*_** were not affected by other immunosuppressive drugs or by antihypertensive agents.

## Discussion

In the present study, we evaluated the spontaneous occurrence of baroreflex and nonbaroreflex episodes in patients with end-stage renal disease on renal replacement therapy and in control subjects. Within this purpose we developed a simple method to estimate SBP-IBI correlations that were evaluated during one-beat overlapping 1 minute epochs time (sliding) windows. This method made possible the assessment of SBP-IBI relationship for longer time epochs, as compared with the analysis of short transient sequences of few cardiac beats in the sequence method. Analysis of overlapping 1 minute epoch time windows also provided different correlations between SBP and IBI than those obtained by the "Z" method that is based on the statistical dependence between SBP and heart rate. The main findings of the evaluation by overlapping 1 minute epochs window methodology were: 1. baroreflex episodes were predominant in Controls and HD, while nonbaroreflex episodes were predominant in TX. A higher proportion of non-baroreflex episodes in TX as compared to HD was also revealed by the "Z" methodology. In contrast, the sequence technique demonstrated a higher proportion of baroreflex than the nonbaroreflex episodes in all groups, independent of renal status (Figure 2). 2. The regression slope (***b*_*sl*_**) of SBP-IBI correlation of both baroreflex and nonbaroreflex episodes was decreased in HD patients as compared to controls; a similar decrease was also detected by other methods. A mild increase of both baroreflex and nonbaroreflex slopes, independent of methodology, was noted in TX (Figure 3). 3. ***b*_*sl*_** was significantly correlated with ***b*_*seq*_** and to a lesser extent to *b*_z_ for each type of patients group (Table 2). The ***b*** coefficients of baroreflex episodes were also significantly correlated with those of nonbaroreflex episodes, when determined by the same methodology.

### Baroreflex and nonbaroreflex episodes in patients on chronic hemodialysis and after renal transplantation

It is generally believed that the control of arterial blood pressure occurs by both feedforward (positive feedback, non-baroreflex) and negative feedback (baroreflex) responses. Thus, sympathetic outflow increases of the arterial pressure via vasoconstriction (feed forward) may be counterbalanced by baroreflex feedback elicited by the elevation in the blood pressure. The preponderance of one mechanism over the other has been debated by several investigators [22,23]. A recent study by Kamiya et al showed that under resting conditions, the sympathetic hemodynamic control is mainly feedforward, while the feedback responses occur in response to active perturbations [24]. Most of the above studies, however, were performed in experimental animals [22,24] or in healthy humans [25]. To our knowledge, the effect of renal function on the type of sympathetic hemodynamic control had not yet been addressed in animals or in human studies. Our data suggest that the cardiovascular regulatory mechanisms modulated by the autonomic nervous system including feedforward (nonbaroreflex) and negative feedback (baroreflex) mechanisms have a different preponderance according to the renal status.

Previous studied performed in healthy individuals using the sequence techniques have shown a higher proportion of baroreflex than that of nonbaroreflex sequences [10,26]. Studies using the "Z" methodology have shown similar proportions of baroreflex and nonbaroreflex in animals with normal renal function; in humans, however, this technique was used only for baroreflex assessment [16,17].

In our study, using the new sliding windows 1 min epochs technique and the less frequently used "Z" methodology, we were able to show a predominance of the baroreflex mediated episodes (feedback) in Controls and also in HD, and of non- baroreflex (feedforward) episodes in TX. For TX patients, the 1 min epochs technique was most discriminatory. The classical sequence technique, which analyses transient variations in blood pressure and heart rate, is based on the presence of short sequences as defined (see Methods); in many cases, even during long term (several hours) recordings, only scarce sequences meeting the above criteria could be found, thus limiting the ability of the technique to differentiate between groups. In contrast, many 1 min epochs with significant (r>0.5) SBP-IBI correlations (the sliding window method), as well as dependent (SBP- heart rate) couples with Z>0 (the "Z" method) could be found in each patient group. Moreover, the 1 min epoch sliding window yields information on SBP- IBI coupling on a longer timescale than the sequence method. The relevance of the timescale in assessing has been pointed out by a previous study that assessed fluctuations in SBP and IBI using both short term (sequence) technique and cross correlation (long term) functions [10]. In that study, it was shown that the sequence method may overlook patterns of negative correlation between SBP and IBI (feedforward) otherwise revealed by analysis performed on larger timescale [10].

The prevailing nonbaroreflex-mediated episodes in TX may suggest a different type of regulation of the arterial pressure in these patients. The nonbaroreflex type of SBP heart rate relationship is believed to be representative of centrally mediated sympathetic activity. This hypothesis is supported by studies performed in experimental animal and in humans that showed that the non-baroreflex activity is abolished by sympathectomy or complete autonomic blockade, but may increase after parasympathetic blockade [2,8,12,16,17,26]. The higher contribution of nonbaroreflex mechanisms in TX patients may be interpreted as evidence for enhanced central sympathetic control. Sympathetic overactivity documented by muscle sympathetic nerve activity recordings (MSNA) was found in TX patients in several studies and was considered to play a role in the pathogenesis of hypertension in these patients [27-29]. In one study, the increase in sympathetic activity in TX patients was not different from that seen in patients on chronic hemodialysis, but was decreased by the removal of the native kidneys. These findings were in agreement with the hypothesis that sympathetic overactivity after transplantation is mediated by signals arising from the diseased kidneys even after reversal of the uremic state [27,28]. Since most of our TX patients were treated with calcineurin inhibitors (Table 1), an attractive hypothesis to explain the increased proportion of non- baroreflex episodes in this group would be sustained exposure to these agents. Nevertheless, the role of calcineurin inhibitors as mediators of sympathetic overactivity and hypertension after renal transplantation is still controversial. Studies in healthy male volunteers have shown that acute administration of cyclosporine, but not of tacrolimus, was associated with an increase in systolic blood pressure and MSNA. Increased blood pressure was also noted after sustained cyclosporine administration, while sustained administration of both cyclosporine and tacrolimus was associated with a decrease in MSNA [29]. In transplanted patients, MSNA as well as plasma norepinephrine level did not change after withdrawal of cyclosporine, despite a significant decrease in blood pressure [28]. The main limitations of the above studies were the relatively small number of patients, and the fact that MSNA data usually are most relevant for the neural domain explored and to a lesser extent to other areas such as the cardiovascular system or the renal circulation [29]. It was suggested that cyclosporine triggers central sympathetic activity by a central pre- or postsynaptic excitation and glutaminergic neurotransmission [30,31]. Renal vascular bed vasoconstriction was proposed to be an additional mechanism of cyclosporine mediated sympathetic activity [30]; this effect, however, might be mitigated by enhanced sodium retention and increased plasma volume [32]. In TX patients, sympathetic activity may be also dependent on other factors, such as antihypertensive medication, the patency of an arterio-venous fistula, the mechanical properties of the arterial wall and the duration of post transplantation follow up [21,33-35]. In our study, the proportion of nonbaroreflex episodes was similar in patients treated with cyclosporine or tacrolimus, and was not affected by antihypertensive medication. We found that the proportion of baroreflex episodes was inversely related to the dialysis vintage in HD, and to time in dialysis prior to transplantation (pre-transplant dialysis vintage) in TX (Table 3). These findings suggest that the baroreceptor contribution to the blood pressure control is preserved in dialysis, but it may diminish with time. Whether the relative decrease in the baroreflex and the increase in the nonbaroreflex contribution are related to alterations in sympathetic signaling from failing kidneys remain to be determined. Prolonged hemodialysis vintage could also have an effect on the relative contribution of baroreflex and nonbaroreflex mechanisms in our transplanted patients (Table 3). These data, however, have to be interpreted with caution, because of the relative small number of TX patients, the large data range and the multitude of factors that affect blood pressure after transplantation.

### SBP-IBI correlations in patients with different renal status

The ***b*** coefficient, i.e. the regression slope of SBP-IBI correlation, is a measure of the strength of blood pressure-heart rate relationship. The ***b*** coefficient of the baroreflex episodes determined by the sequence technique (*b*_*seq*_) representing the magnitude of the heart rate response to spontaneous blood pressure changes, is one of the most commonly used methods of baroreflex sensitivity determination and is strongly affected by the renal function [21,36]. In agreement with previous reports, in the present studies, baroreceptor ***b*_*seq*_**, ***b*_*sl*_** and *b*_z_ are decreased in HD patients, and restored at, least partly in TX [21]. *b*_*seq*_ of baroreflex episodes was significantly correlated with ***b*** coefficient determined by 1min sliding windows epoch (***b*_*sl*_**), and to a lesser extent to *b*_z_ (Table 2). The latter finding is similar to those of previously reported studies in which the "Z" method exhibited lower agreement with other techniques of baroreflex sensitivity determination [15]. Similar to the baroreflex ***b*** coefficients, the nonbaroreflex *b*_*seq*_, ***b*_*sl*_** and *b*_z_ were decreased in HD, but comparable to those of controls in TX patients. When the same technique was used, the *b*_z_ coefficients of baroreflex episodes significantly correlated to the *b*_*seq*_, ***b*_*sl*_** and *b*_z_ coefficients of non-baroreflex episodes (not shown in table). Both types of ***b*** slopes were significantly correlated to α coefficients and negatively correlated to age (Figure 5), independent of renal status. Taken together, these findings may suggest a similar dependency of both baroreflex and nonbaroreflex episodes on the autonomic nervous system and a common effector mechanism. In hemodialysis patients both baroreflex and nonbaroreflex ***b*_*sl*_** were inversely correlated to CRP, confirming previous observations on the connection between inflammatory markers and autonomic nervous system dysfunction in severe renal insufficiency [37,38].

In TX patients, the finding of greater ***b*** slope (*b*_*seq*_ and ***b*_*sl*_**) of nonbaroreflex episodes in patients treated with cyclosporine may support previous observations [29] that showed differences between cyclosporine and tacrolimus on modulation of the sympathetic activity. Nevertheless, our data obtained from patients with renal insufficiency are difficult to compare with those of the former study which was performed in healthy individuals and used a different methodology [29].

### Limitations

The main shortcoming of our study is the use of noninvasive methodology of assessment of autonomic nervous function, since MSNA studies were not available or not-applicable to the majority of our patient population. Because of the relative small number of transplanted patients, only limited comparisons of specific effects of drugs, such as cyclosporine versus tacrolimus, could be performed. Our study patient population included many elderly individuals with multiple comorbidities. These patients however, are most characteristic of the present day hemodialysis and even transplant population.

## Conclusions

Using the new 1 min epochs sliding windows method as well as conventional techniques, we showed that renal status may affect the distribution of baroreflex and nonbaroreflex activity, as well as the strength of systolic blood pressure-heart rate relationship. Baroreflex sequences are more frequent in hemodialysis patients, while nonbaroreflex episodes are most frequently encountered in transplanted patients.

The predominant nonbaroreflex contribution may suggest enhanced sympathetic control in these patients. In hemodialysis patients, both baroreflex and nonbaroreflex ***b*** coefficients, representative of the magnitude of heart rate response to blood pressure oscillations, are significantly decreased and inversely correlated to the inflammatory marker CRP. In transplanted patients, this decrease is at least partially reversed, and seems to be related to the type of calcineurin inhibitor. Our data may be relevant for understanding of the pathogenesis and selection of appropriate treatment of post-transplant hypertension.
